# Estimating production costs and retail prices in different poultry housing systems: conventional, enriched cage, aviary, and barn in Japan

**DOI:** 10.1016/j.psj.2022.102194

**Published:** 2022-09-21

**Authors:** H. Kato, Y. Shimizuike, K. Yasuda, R. Yoshimatsu, KT. Yasuda, Y. Imamura, R. Imai

**Affiliations:** ⁎ Animal Welfare and Wildlife Damage Management Group, National Agriculture and Food Research Organization, Tsukuba 305-0901, Japan; † Research Faculty of Agriculture, Hokkaido University, Sapporo 060-8589, Japan; ‡ Hytem Co., Ltd, Kakamigahara City, 509-0109, Japan; § Graduate School of Agriculture, Hokkaido University, Sapporo 060-8589, Japan

**Keywords:** animal welfare, poultry production system, production cost, retail price, Japan

## Abstract

For many producers, introduction of improved animal welfare systems is a turning point in their future production strategies as it increases production costs. The increase in egg retail prices is of growing concern not only for producers, but also for retailers and consumers. However, no report has calculated the estimated production costs or retail prices associated with introducing practices that support improved animal welfare in poultry farms in Japan. Therefore, this study aimed to estimate the production costs and table egg prices of 6 types of laying hen systems: conventional cage (**CC**): 8- and 12-tiers (**CC8, CC12**), enriched cage (**EC**): 8- and 12-tiers (**EC8, EC12**), aviary (**AV**), and barn systems (**BR**). Production costs include land purchases, construction costs of facilities, equipment and machinery, quantity of feed provided, farming materials invested, and wages.

As a result, farm gate prices were estimated as CC8 = 12.19, CC12 = 12.19, EC8 = 14.52, EC12 = 14.52, AV = 21.14, and BR = 28.74 [yen/egg], and the production cost, including building the new farm, increased by EC8 = 19.1%, EC12 = 19.1%, AV = 73.4%, and BR = 135.7%, respectively, referring to the value of CC. The results show that the prices increase in systems between CC and BN. The retail price or table egg price was estimated to be CC8 = 24.68, CC12 = 24.68, EC8 = 28.07, EC12 = 28.07, AV = 37.27, and BR = 48.53 [yen/egg]. The retail price of BR is approximately twice that of CC. In addition, assuming that all of Japan's eggs were produced in the BR system, the soaring cost of eggs would likely affect the prices of factory eggs, such as liquid eggs and other products, thus affecting the prices of various food products.

Understanding the significant management costs that affect the retail price of eggs would facilitate improved policies and practical approaches to support poultry farms and sustainable farming activities while addressing public concerns.

## INTRODUCTION

In Japan, the Act on the Welfare and Management of Animals (Ministry of the Environment (**MOE**, 1973) was enacted in 1973, based on which the Standards Relating to the Care and Keeping of Industrial Animals was announced in 1987 (MOE, 1987). These standards require industrial animals to be in good physical and psychological conditions. Good animal welfare is considered essential to reduce stress and disease in livestock; raising them in a comfortable environment can lead to improved productivity and production reliability of livestock products (Ministry of Agriculture, Forestry and Fisheries (**MAFF**), 2022a). Based on this concept, in 2011, the Japan Livestock Technology Association (**JLTA**, (2011) compiled the Livestock Management Guidelines for the Concept of Animal Welfare, promoting animal welfare and educating people involved in livestock production. However, there are no legal animal welfare regulations yet, and animal conditions are left to farmers’ initiatives. As a result, according to the second version of the Animal Protection Index created by World Animal Protection (2020), Japan received an evaluation of the lowest rank, “G,” for the protection of animals used in farming. This is perhaps unsurprising given that the Japanese livestock farming industry has only recently started to discuss whether to adopt an animal welfare system in livestock management systems. In Japan, the term “farm animal welfare” is not well known to consumers, with 70% of them not knowing the term (Shiga et al., 2020).

Several European countries are making continuous efforts to improve their animal welfare laws, and intergovernmental organizations are actively engaged in related activities. Conventional cage systems for laying hens were banned in the European Union in 2012 (EU Directive 1999/74/EC). Additionally, the non-cage system became the standard production system in the EU (Mench et al., 2011).

In contrast, according to a survey by JLTA (2014), 92% of egg-laying chicken-keeping systems in Japan are produced using conventional cages, and approximately 60% of farmers recognize animal welfare guidelines. Approximately 60% of the farmers indicated that they would consider altering the conventional system to an alternative system in the future. The average annual Japanese domestic egg production in 2020 was 2.64 million tons per year, 53% of which was for household consumption, and the self-sufficiency rate was high at 96%. Average egg imports were 106,000 tons per year, primarily eggs in powder form, accounting for 4% of domestic consumption. Exports were low, at approximately 18,118 tons per year (Japan Poultry Association, 2021). Egg consumption was 340 eggs per capita per annum, the second largest in the world (International Egg Commission, 2020). The average retail price from 2011 to 2021 was 206 ± 15 yen for 10 eggs (MAFF, 2021a), and eggs were regarded as an inexpensive staple food. For many producers, the introduction of improved animal welfare systems is a turning point in their future production strategies that increases production costs. This cost increase is expected to be passed on to consumers with higher retail prices. Some analyses on price fluctuations and economic aspects of the introduction of keeping animals in higher welfare conditions have been conducted outside Japan. Data from California indicate that shifting from conventional cages to barn housing would likely cause farm-level cost increases of approximately 40% per dozen (Sumner et al., 2011). Brannan and Anderson (2021) showed a 36% increase in labor cost per hen in cage-free vs. cage system. Daniel et al. (2008) found that non-cage production systems had production costs 20% higher than conventional cage production, and stated that the average retail price of non-caged eggs was often double of that of conventional eggs. The increase in egg retail prices is of growing concern not only for producers, but also for retailers and consumers. However, no report has calculated the estimated production costs or retail prices associated with introducing practices that support improved animal welfare in poultry farms in Japan. Therefore, this study aimed to estimate the production costs and table egg price of 4 types of laying hen systems: conventional cage, enriched cage, aviary, and barn systems. Production costs include land purchases, construction costs of facilities, equipment and machinery, quantity of feed provided, farming materials invested, and wages. Understanding the significant management costs that affect the retail price of eggs would facilitate improved policies and practical approaches to support poultry farms and sustainable farming activities while addressing public concerns.

## MATERIALS AND METHODS

### The Setting of Farm Size and Research Framework

Japan has approximately 2,000 poultry farms, and there has been a push to consolidate, mechanize, and scale-up poultry farming operations to improve productivity. Approximately 330 poultry farmers maintained more than 100,000 birds per farm and accounted for 80% of the total number of egg-laying hens in Japan (MAFF, 2008, 2021b). If those poultry farmers changed their laying system from conventional cages to systems with improved animal welfare, this would significantly impact the public.

This study established a virtual poultry farm that kept approximately 110,000 birds per farm and was located 50 to 100 kms away from a feed factory near a port area. Production costs were calculated by estimating land purchase costs, facility construction, equipment costs, purchase cost of pullets at 120 d of age, feed, labor, other management costs, sold spent hen compensation cost, and gross profit. Egg production efficiency was obtained from the farm records. Egg production was estimated during one cycle, from all-in to all-out, for the number of birds stored in the house on the farm. Simultaneously, the retail price was calculated by accumulating the costs and profits associated with the Grading and Packing (**GP**) center, wholesaler profits, and retailer profits.

### Overview of Surveyed Farmers Related to Production Efficiency

In this study, the production efficiency of egg-laying hens was investigated for farmers with facilities capable of producing more than 100,000 birds per farm. The average facility hen capacity of the 8 surveyed farms was 1,151,168 (±1,281,457) birds per farm. The surveyed farms owned multiple types of hen houses, such as conventional cage houses and houses with higher animal welfare standards, and the average productivity units or other information for each house were obtained. Our target houses were 14 houses; 7 conventional cage (**CC**) houses, 3 enriched cage (**EC**) houses, 3 aviary (**AV**) houses and one barn (**BR**) house. When data could not be obtained for various reasons or the unit value was not representative of the average, academic papers or personal communication was used. Additionally, feed has significant ingredients of the feed differences for each farm, and feed and pullet also have significant price differences in proportion to the purchased quantity. More realistic and versatile figures were calculated with reference to the statistical data. The opinions of experts in the field were sought to determine the appropriateness of the data. For clarity, the results of this study were converted into units per egg or 10 eggs.

The survey items and production efficiency of egg-laying hens are shown in Table 1. We defined the age when entering the housing as 120 d and the age at 80% egg productivity as 180 d. The conventional system forced molding for 30 d, whereas the improved animal welfare systems were assumed to have none. The other values are the average values obtained from the farms, but some values are set as follows: the age at end of lay of the BR were modified to valid values (personal communication; feed company A, March 1, 2022). The egg weight of the CC was calculated by including the figures from Ghen Corporation (2014), Goto (2020), and farm records (n = 8). The floor-egg ratio of the BR was the average of the survey data (n = 1) and data from Noguchi et al. (1999). Average egg weight [g/egg], livability at end of lay [%], feed intake [g/birds/day], and feed conversion of BR were cited by Noguchi et al. (1999). A survey on production efficiency was conducted from September 2021 to February 2022.

**Table 1:** Survey items and production efficiency of egg-laying hens.

Hen houses type		CC	EC	AV	BR
Item / Hen type	Unit	Julia-Lite (LSL-Lite)	Boris Brown (Hy-Line Brown)	Boris Brown (Hy-Line Brown)	Boris Brown (Hy-Line Brown)
Age at transferred to laying house*1	day	120.0	120.0	120.0	120.0
Forced molding⁎2	-	yes	-	-	-
Age at 80% egg-productivity*3	day	180.0	180.0	180.0	180.0
Age at end of lay	day	700.0*4	526.0*5	560.0*6	543.0*7
Term of egg-laying (TEL) *8	day	490.0*9	346.0	380.0	363.0
Average of egg-laying ratio*10	-	87.3*11	87.1	87.7	85.0
Average of egg-weight	g/egg	62.6*12	63.0*13	60.0*14	62.8*15
Number of eggs-laying*16	egg/TEL /birds	427.8	301.4	333.3	308.6
Average of egg weight*17	kg/TEL /birds	26.8	19.0	20.0	19.4
Livability at end of lay*18	-	91.4	95.0	90.3	85.4*19
Undergrade egg ratio⁎20	-	9.5	8.7	13.5	12.0
Floor egg ratio	-	-	-	2.3*21	2.0⁎22
Feed intake*23	g/birds/day	106.0	107.5	115.0	132.9*24
Feed conversion*25	-	2.0	2.1	2.3	2.4*26

*1 The values set in this study.

⁎2 The values set in this study.

*3 The values set in this study.

*4 The values set in this study.

*5 Average of surveyed values (n = 3).

*6 Average of surveyed values (n = 2).

*7 The values set in this study.

*8 EC, AV, BR = age at end of lay[days]-age at 80% egg-productivity[days].

*9 CC = age at end of lay[days]-age at 80% egg-productivity[day]-30[days].

*10 Average of surveyed values (n = EC:3, AV:3, BR:1).

*11 Average of surveyed values, Ghen co. (2014) and Goto et al. (2020) (n = CC:8).

*12 Average of surveyed values, Ghen co. (2014) and Goto et al. (2020) (n = CC:8).

*13 Average of surveyed values (n = EC:3).

*14 Average of surveyed values (n = AV:3).

*15 Noguchi et al. (1998).

*16 CC, EC, AV, BR = TEL [days] × ( average of egg-laying ratio [%]/100).

*17 CC, EC, AV, BR = Number of eggs-laying[egg] × average of egg-weight[g/egg].

*18 Average of surveyed values (n = CC:3, EC:3, AV:3).

*19 Noguchi et al. (1998).

⁎20 Average of surveyed values (n = CC:4, EC:3, AV:2, BR:1).

*21 Average of surveyed values (n = AV:3).

⁎22 Average of surveyed values and Noguchi et al. (1998) (n = BR:2).

*23 Average of surveyed values (n = CC:6, EC:2, AV:3).

*24 Noguchi et al. (1998).

*25 Average of surveyed values (n = CC:7, EC:2, AV:2).

*26 Noguchi et al. (1998).

The formulae are shown below.

Term of egg-laying (**TEL**) (with forced molding) [days] = Age at end of lay [days] - Age at 80% egg-productivity [days] - 30 [days].
TEL (without forced molding) [days] = Age at end of lay [days] - Age at 80% egg-productivity [days].
Number of hens (at end of lay) [birds] = Number of hens (transferred to laying house) [birds] × (livability at end of lay [%] / 100)
Average number of hen laying [birds] = (number of hens (transferred to laying house) [birds] + number of hens (at end of lay) [birds]) / 2
Number of eggs [egg /TEL] = Average number of hen laying [birds] × (average egg-laying ratio [%] / 100) × TEL [days]
Number of undergrade eggs [egg / TEL] = Number of eggs [egg/TEL] × ( (undergrade egg ratio (cracked, shell-less, dirty eggs, etc.) [%] + floor egg ratio [%]) / 100)
Salable egg [egg / TEL] = Number of eggs [egg/TEL] − Number of undergrade eggs [egg/TEL]
Production egg weight [kg/ TEL] = Salable eggs [egg/TEL] × average egg weight [g/egg]

### Types of Chicken

In the field survey, all cases produced white eggs in conventional cages and brown eggs in the animal welfare production system. The share of foreign breeder chickens is 96% in Japan (MAFF, 2019b). Generally, the white eggshell chicken breed has a high proportion of Julia-Lite (LSL-Lite), and the brown eggshell chicken breed has a high proportion of Boris Brown (Hy-Line Brown). Therefore, we set parameters as Julia-Lite (LSL-Lite) for conventional cages and Boris Brown (Hy-Line Brown) for the alternative system.

### Cost of Building the Facility and Purchasing the Land

Changing the system based on the area per unit of birds from traditional housing to an alternative system was a primary need to comply with improved animal welfare standards. Conventional cages’ size in Japan typically ranges from 370 to 430 [cm^2^/birds] (JLTA, 2014). To change the system, hen houses would need to be renovated or transformed into newly constructed facilities, which would require significant investment and is one of the biggest concerns for farmers. In addition, a decrease in the number of hens per unit area will require an increased facility and land area for the same number of laying hens. Thus, a change from conventional cages requires not only a change in facilities, but also poses a significant land acquisition issue. Therefore, in this study, farm building costs were calculated based on the assumption that new land was purchased and a new facility was constructed.

Table 2 describes the poultry hen houses used in this study. This study sets up a facility capable of maintaining approximately 110,000 bird houses that were constructed with a steel framework and sandwich panels. There are 6 types of hen houses: conventional cage 8-tier (**CC8**), conventional cage 12-tier (**CC12**), enriched cage 8-tier (**EC8**), enriched cage 12-tier (**EC12**), aviary (**AV**), and barn (**BR**). Costs were classified into 2 categories: land purchase costs and poultry housing prices. The purchased land area included the building area, the area required for the construction of ancillary facilities, such as egg collection rooms and manure management facilities, and a buffer area. The purchase cost of land included the cost of land, land development, primary electrical work, and primary water supply and drainage. The price of a hen house consists of facility construction and equipment costs. Facility construction cost is the cost of constructing the main building. The cost includes the main facility's price as cages, peripheral equipment such as ventilation fans, a manure conveyor, an egg collection conveyor, a set of electric panels in the hen house, and the costs of domestic freight, installation, and electric and drainage construction. However, egg collection buildings and poultry manure management facilities were not included. Facility construction and land purchase costs were calculated with a depreciation period of 17 yr. The survey year of 2021 was chosen for the cost of building the facility and purchasing the land. The value of 100 Japanese yen (USD 0.74) on June 23, 2022, was used for the estimation.

**Table 2:** Description of the poultry hen house.

Type of hen			Julia-Lite (LSL-Lite)	Julia-Lite (LSL-Lite)	Boris Brown (Hy-Line Brown)	Boris Brown (Hy-Line Brown)	Boris Brown (Hy-Line Brown)	Boris Brown (Hy-Line Brown)
Item		Unit	CC8	CC12	EC8	EC12	AV	BR
General description	Total number of houses on the farm	house/farm	1	1	1	1	1	16
	Total number of layers of the house	birds/house	112,320	112,320	114,540	113,832	111,472	7,203
	Total number of layers per farm	birds/farm	112,320	112,320	114,540	113,832	111,472	115,248
	House site length × width	m	18.0 × 93.0	13.0 × 93.0	25.8 × 106	20.0 × 96.8	36.4 × 89.3	12.8 × 75.0
	House area	m^2^	1,674	1,209	2,735	1,936	3,250	960
	Housing area per farm	m^2^	1,674	1,209	2,735	1,936	3,250	15,360
	Total number of layers par house area	birds/m^2^/farm	67.1	92.9	41.9	58.8	34.3	7.5
	The land area required with ancillary facilities*1	m^2^/farm	9,792	8,960	10,880	10,240	11,602	46,000
	The total area of the farm including a buffer area	m^2^/farm	12,729	11,648	14,143	13,311	15,082	68,996
Room's description	Number of rooms in one house	room	2	2	2	2	4	1
	Standard capacity per room	birds/room	56,160	56,160	57,120	56,916	27,868	7,203
	Rows	-	3	2	4	3	3	2
	Tiers	-	8	12	8	12	-	-
	Length of system	m	79.3	79.3	86.8	76.9	82.8	66.7
	Layers per cage	birds/cage	9	9	51	51	-	-
	Living space per bird	cm^2^/birds	432	432	758	758	1,111	1,111

*1 Ancillary facilities are manure management building, egg collection room etc., and those costs are not included facility's price in this study.

### Number of Farm Workers and Working Hours

In recent years, daily egg-laying chicken production has become increasingly automated, and the number of farm workers and working hours have decreased markedly. In contrast, AV and BR systems have increased the number of workers and working hours owing to their facility structures. The number of workers and working hours in the 4 types of facilities in this study were calculated from farm records and corrected per 110,000 birds per farm. However, regarding the BR system, 16 buildings were required to house 110,000 birds, and it was determined that each building was managed by one worker per building (7,203 housing birds per worker).

### Purchase of Pullet at 120 Days Age

In this study, all systems purchased pullets at 120 d of age from a chick brooding company. Since the price of pullets at facilities with more than 100,000 birds deviated from statistical figures, the cost per pullet for CC and EC pullets at 120 d of age was calculated using the 2016–2019 average price of 933 [yen/chick] from MAFF (2020c), with a corrected to a fairer price of 839 [yen/chick] (personal communication; Feed company B, December 23, 2021). The AV and BR systems need to be trained to fly pullets at 120 d of age in a system with animal welfare. We interviewed chick brooding company A (December 23, 2021), regarding this price, and we conclude the following: a chick brooding company must renovate or build a new chick house to take care of trained chicks. The cost of debeaking newly hatched chicks is expected to be higher because of the use of an infrared beak treatment. The price of trained pullets is expected to be approximately 1.5 times higher than the regular price because of the economic investment required by a chick brooding company. As such, we set the price of trained pullets to 1,300 [yen/chick], which is approximately 1.5 times the standard price of pullets.

### Percentage of Feed Costs to Production Costs, Feed Costs, and Transportation Costs

Feed costs account for the highest percentage of production costs for egg-laying hens, and are an important factor in production costs. In this study, the feed, production, and feed transportation costs were examined. Table 3 shows the ratios of feed cost and other expenses to production cost; CC and EC are the average values from the farm records, and AV and BR were determined based on farm records and personal communications (feed company A, March 1, 2022).

**Table 3:** Ratio of feed costs and other expenses to production costs.

Facilities	Unit	CC	EC	AV	BR
Feed	[%]	65.0*1	57.5⁎2	45.0*3	40.0*3
Other expenses	[%]	35.0*1	42.5⁎2	55.0*3	60.0*3

*1 Average of surveyed values (n = CC:3).

⁎2 Average of surveyed values (n = EC:2).

*3 The values set in this study by personal communication; Feed company A, Mar 1, 2022

In Japan, imported corn is the main feed source for poultry farms. However, nowadays, farmers have several other choices for expanding the production and utilization of domestic feed resources, or for one of the consumer's food choices. Table 4 shows the compositions of the feed ingredients used in this study. The basic feed was based on 58% imported corn as the main ingredient; substitutions of domestic feed rice, domestic corn, and imported non-GM corn as the other main ingredients were also examined. Table 5 shows the feed and feed transportation costs (Japan Compound Feed Supply and Stabilization Organization, 2021), personal communication (feed company B, December 23, 2021; feed company A, March 1, 2022). Transportation costs were set at a distance of 50 to 100 kms from the feed company to the farm (MAFF, 2020b).

**Table 4:** Composition of the feed ingredients.

Ingredients	Basal diet [%]
Corn grain	58
Protein modified foods	30
Feed additives	12

**Table 5:** Feed costs and feed transportation costs.

Item	Unit	Feed unit price	Freight (50–100 km)	Total feed unit price
Imported corn	yen/t	46,048.8^a^	2,671.0^c^	48,719.8
Domestic rice feed	yen/t	44,998.8^b^	2,671.0^c^	47,669.8
Domestic corn	yen/t	49,698.8^b^	2,671.0^c^	52,369.8
Imported non-GMO corn	yen/t	58,348.8^b^	2,671.0^c^	61,019.8

a Japan Compound Feed Supply and Stabilization Organization (2021).

b Personal communication; Feed company B, Dec 23, 2021, Feed company A, Mar 1, 2022.

c MAFF (2020b).

### Income From Spent Hens and Compensating Expenses

In general, spent hens are purchased at a fair price by the spent chicken supplier. In this study, the average purchase price for CC and EC (n = 9), 16.5 yen per spent hen, was established as the selling price of the spent hens for CC and EC. However, for AV and BR, the sale price was zero yen in 2 out of 4 cases. The main reason was the time and effort required to capture egg-laying hens while taking the spent hens out of the house. As such cases will likely increase in the future when the animal welfare system increases, this study sets the sale price for AV and BR at zero. In addition, the amount that would typically be the income from the sale of spent hens (AV and BR: 0.06 yen per egg production) was added to the production cost to compensate for expenses.

### Hired Labor and Other Running Expenses

According to MAFF (2019a), for operations with 30,000 or more birds, labor costs accounted for 30% of the total costs of farming operations minus the costs of purchased pulletss, feed, farm equipment, and facilities. This value was used in the estimation. The labor cost was divided by the number of workers in each system. Since the labor cost of BR was significantly lower than that of other systems, it was adjusted upward to the level of 4.1 million yen per worker, the average labor cost of CC, EC, and AV.

Other expenses, such as utilities, were defined as the remainder of the other expenses to the cost of production minus the price of purchased pullets and the cost of feed, facilities, and labor (Table 3).

### Gross Profits

Gross profit was assumed to be constant in all systems and was 8% of the add-up feed cost and other expenses (personal communication, retail company, March 1, 2022)

### GP Center Expenses and Profits, and Wholesaler and Retailer Profit Margins

In Japan, where it is customary to eat raw eggs, considerable attention is paid to egg hygiene. Eggs shipped from the farm are cleaned and packed at the GP center. The number of bacteria on the eggshell is reduced by an average of 1/10 to 1/100 after washing (Imai, 1983). This study assumed that all salable eggs produced were processed at the GP center and shipped for table eggs. We used the average of 5 farms owning GP centers for GP center expenses, which was 29 [yen/kg]. The GP center's profit was assumed to be 50 [yen/kg] (personal communication; agricultural cooperative, December 28, 2021). Wholesaler and retailer profits were assumed to be the retail price with a 44% profit on the GP center shipping price (personal communication; retail company, March 1, 2022).

## RESULTS AND DISCUSSION

### Egg Production

Egg production of each system is shown in Table 6. Several studies have identified differences in the number of eggs and egg weights in different facility systems (Abrahamsson and Tauson, 2009; Ahammed et al., 2014). In the estimates of this study, the number of eggs produced decreased by 26.2, 26.6, 28.5, and 31.9% for EC8, EC12, AV, and BR systems, respectively, referring to the value of CC as 100. Compared with the CC system, the egg weight decreased by 25.7, 26.1, 31.4, and 31.7% in EC8, EC12, AV and BR systems, respectively. The production efficiency of AV and BR was lower due to their shorter production term, higher undergrade egg ratio, such as dirty eggs, shell-less eggs, cracked eggs of approximately 13%, and higher floor-egg ratio of approximately 2% (Table 1). This decrease in production performance has a significant impact on retail price.

**Table 6:** Poultry egg production for each system.

Item	Unit	CC8	CC12	EC8	EC12	AV	BR
Number of salable eggs	10^3^ egg/TEL	41,612.9	41,612.9	30,727.5	30,537.5	29,762.5	28,349.0
Weight of salable egg	t /TEL	2,605.0	2,605.0	1,935.8	1,923.9	1,785.8	1,780.3

Abbreviations: AV, aviary; BR, barn; CC8, conventional cage 8-tier; CC12, conventional cage 12-tier; EC8, enriched cage 8-tier; EC12, enriched cage 12-tier; TEL, term of egg-laying.

Salable egg excludes undergrade eggs (cracked, shell-less, dirty eggs, etc.).

### Cost of Building the Facility and Purchasing the Land, Number of Hens Per Facility and Farmland

Table 7 shows the costs of building a facility and purchasing the land. The results show that prices increase in systems from CC to BR. Particularly, BR requires 16 hen houses to manage approximately 110,000 laying hens. Therefore, the required poultry house costs were CC8 = 3,036, CC12 = 2,917, EC8 = 4,681, EC12 = 4,496, AV = 5,990, and BR = 13,565 [yen/birds/house]. In addition, the farm construction cost, including building the facility and purchasing the land was CC8 = 520, CC12 = 510, EC8 = 740, EC12 = 700, AV = 910, and BR = 2,500 [million yen / farm]. This implies that cost was:CC8 = 4,699; CC12 = 4,498; EC8 = 6,429; EC12 = 6,189; AV = 8,139; BR = 21,693 [yen/birds/farm]. Animal welfare requires a large initial investment in the construction of buildings, and it would be a significant financial burden for farmers to pay the initial investment themselves.

**Table 7:** Cost of building the facility and purchasing the land.

Item		Unit	CC8	CC12	EC8	EC12	AV	BR
Item / Hen house cost	Hen house equipment	10^3^ yen/ house	141,424	144,502	237,974	243,446	202,278	13,884
	Hen house peripheral equipment*1	10^3^ yen/ house	19,451	19,430	24,169	23,819	28,951	7,873
	Freight, installation of the equipment, electrical wiring and water piping	10^3^ yen/ house	53,567	54,045	67,185	68,827	141,442	12,073
	Hen house building construction	10^3^ yen/ house	126,600	109,700	206,800	175,700	295,000	63,881
	Total hen house cost per house	10^3^ yen/ house	341,042	327,677	536,128	511,792	667,670	97,712
	Total hen house cost per farm	10^3^ yen/ farm	341,042	327,677	536,128	511,792	667,670	1,563,393
	Total hen house cost per birds per farm	yen/ birds/ farm	3,036	2,917	4,681	4,496	5,990	13,565
Land area cost	Land cost	10^3^ yen/ farm	19,252	17,617	21,392	20,133	22,812	104,356
	Land development costs	10^3^ yen/ farm	88,857	81,309	98,730	92,922	105,285	417,423
	Primary electricity and drainage work	10^3^ yen/ farm	78,624	78,624	80,178	79,682	111,472	414,893
	Total land purchase cost	10^3^ yen/ farm	186,733	177,550	200,300	192,738	239,569	936,672
Total cost for poultry farm construction		10^3^ yen/ farm	527,776	505,227	736,428	704,530	907,239	2,500,065
Total cost for poultry farm construction per birds per farm		yen/ birds/ farm	4,699	4,498	6,429	6,189	8,139	21,693

Abbreviations: AV, aviary; BR, barn; CC8, conventional cage 8-tier; CC12, conventional cage 12-tier; EC8, enriched cage 8-tier; EC12, enriched cage 12-tier.

*1 Hen house peripheral equipment were ventilation fans etc. One house per one farm based on this theoretical comparison study. When by BR, one farm consists of 16 houses.

The required area for managing laying hens is presented in Table 8. The required poultry living spaces were CC8 = 432, CC12 = 432, EC8 = 758, EC12 = 758, AV = 1,111, and BR = 1,111 [cm^2^/bird]. The farm area was CC8 = 1,133; CC12 = 1,037; EC8 = 1,235; EC12 = 1,169; AV = 1,353; BR = 5,987 [cm^2^ /birds/farm]. The farm area was 1.2 to 5.4 times larger than the required living space. If all the laying hens in Japan (140 million birds; MAFF 2021b) in each system occupied the total area of cultivated land in Japan (Ministry of Land Infrastructure Transport and Tourism, 2022), the CC8, CC12, EC8, EC12, AV, and BR would occupy 0.037, 0.033, 0.040, 0.038, 0.044, and 0.193% of cultivated land, respectively. The results show that changing to an animal welfare system requires a change in facilities, equipment, and land.

**Table 8:** Required area for managing laying hens.

Item	Unit	CC8	CC12	EC8	EC12	AV	BR
Living space per bird	cm^2^/birds	432	432	758	758	1,111	1,111
The total farm area allotment for birds	cm^2^/birds	1,133	1,037	1,235	1,169	1,353	5,987
The total farm area allotment for birds in Japan	ha	1,595	1,460	1,738	1,646	1,905	8,428
Raito of poultry farm area to total farmland in Japan	%	0.037	0.033	0.040	0.038	0.044	0.193

Abbreviations: AV, aviary; BR, barn; CC8, conventional cage 8-tier; CC12, conventional cage 12-tier; EC8, enriched cage 8-tier; EC12, enriched cage 12-tier.

### Number of Farm Workers and Working Hours

The number of workers managing 110,000 birds was CC = 3.1, EC = 2.8, AV = 7.4, and BR = 16 [worker/110,000 birds/day]. Working hours were 1.4, 2.0, 1.6, and 3.4 [hours/worker/110,000 birds/day], for CC, EC, AV, and BR, respectively.

According to these findings, the EC system has fewer workers and higher working hours than the CC system. Our calculations were based on the farm records. The poultry management system has a variety of functions and we might have missed some when counting these items. Research on each work hour and the number of workers on a poultry farm in detail will be our future work. The improved animal welfare system increases the number of farm workers and working hours required. The average age of farm workers mainly engaged in farming was 67.8 yr, and the population of people mainly engaged in farming in Japan decreased from approximately 3.4 million in 1995 to approximately 2.2 million in 2018 (MAFF, 2018, 2020d). Moreover, to promote animal welfare, the securing of farm workers must also be considered. In the future, it will be necessary to evaluate the agricultural workload from the perspective of farmers’ welfare.

### Table Egg Retail Prices by Type of Hen house

#### Production Cost in Farm and Farm Gate Price

Table 9 shows poultry egg production cost and table egg price. The highest production cost on the farm was feed costs but the share of these costs varied by production system: in BR, the share of feed costs was relatively lower than in other systems because of the higher share of facility costs. Additionally, 16 workers were needed to manage this system; the labor cost ratio (9%) was higher than in other production systems. From an economic standpoint, because the BR production system requires a large amount of labor and staff wages, significant difficulties can be expected in conducting large-scale production. The cost burden associated with the free take-back of spent hens was only 0.06% of total expenses in AV and BR and had a minimal impact on production costs. Farm construction costs were 6.3 to 19.3% of total production costs. Financial support to a farm in the form of a subsidy may lead to a reduction in production costs. As a result, farm gate prices were estimated as CC8 = 12.19; CC12 = 12.19; EC8 = 14.52; EC12 = 14.52; AV = 21.14, and BR = 28.74 [yen/egg], and the estimated production cost increased by 1.2, 1.2, 1.7, and 2.4 times, for EC8, EC12, AV, and BR, respectively, referring to the value of CC as 1. Previous studies showed that non-cage production systems had production costs 20 to 40% higher than conventional cage production (Daniel et al. 2008; Sumner at al. 2011). The scales used to estimate production cost in this study were different from those used in the earlier study; however, similar results were obtained for the animal welfare level relationship, with increased production cost for EC8 = 19.1%; EC12 = 19.1%; AV = 73.4% and BR = 135.7% for including building the new farm.

**Table 9:** Poultry egg production cost and table egg price.

Category	Item	Unit	CC8	(%)	CC12	(%)	EC8	(%)	EC12	(%)	AV	(%)	BR	(%)
Farm	Farm construction cost	yen/egg	0.75	(6.6)	0.71	(6.3)	1.41	(10.5)	1.36	(10.1)	1.79	(9.2)	5.19	(19.4)
	Pullet at 120 days cost	yen/egg	2.26	(20.1)	2.26	(20.1)	3.13	(23.3)	3.13	(23.3)	4.87	(24.9)	5.28	(19.8)
	Feed	yen/egg	7.34	(65.0)	7.34	(65.0)	7.73	(57.5)	7.73	(57.5)	8.79	(44.9)	10.32	(38.7)
	Labor cost	yen/egg	0.28	-	0.29	-	0.35	-	0.37	-	1.22	-	1.50	-
	Adjusted labor cost	yen/egg	0.00	-	0.00	-	0.00	-	0.00	-	0.00	-	0.81	-
	Subtotal labor cost	yen/egg	0.28	(2.5)	0.29	(2.6)	0.35	(2.6)	0.37	(2.7)	1.22	(6.2)	2.31	(8.7)
	Other production cost	yen/egg	0.66	(5.8)	0.68	(6.0)	0.82	(6.1)	0.86	(6.4)	2.85	(14.6)	3.51	(13.1)
	Sold spent hens compensation cost	yen/egg	0.00	(0.0)	0.00	(0.0)	0.00	(0.0)	0.00	(0.0)	0.06	(0.3)	0.06	(0.2)
	Subtotal production cost	yen/egg	11.29	(92.6)	11.29	(92.6)	13.44	(92.6)	13.44	(92.6)	19.58	(92.6)	26.67	(92.8)
	Profit	yen/egg	0.90	(7.4)	0.90	(7.4)	1.08	(7.4)	1.08	(7.4)	1.56	(7.4)	2.06	(7.2)
	Farm gate price	yen/egg	12.19	(100.0)	12.19	(100.0)	14.52	(100.0)	14.52	(100.0)	21.14	(100.0)	28.74	(100.0)
GP center	GP center expenses	yen/egg	1.82	(36.7)	1.82	(36.7)	1.83	(36.7)	1.83	(36.7)	1.74	(36.7)	1.82	(36.7)
	GP center profit	yen/egg	3.13	(63.3)	3.13	(63.3)	3.15	(63.3)	3.15	(63.3)	3.00	(63.3)	3.14	(63.3)
	GP center gate price	yen/egg	17.14	(100.0)	17.14	(100.0)	19.49	(100.0)	19.49	(100.0)	25.88	(100.0)	33.70	(100.0)
Wholesale/retailer	Profit ratio	%	44.00	-	44.00	-	44.00	-	44.00	-	44.00	-	44.00	-
Retailer	Retail price	yen/egg	24.68	-	24.68	-	28.07	-	28.07	-	37.27	-	48.53	-

Abbreviations: BR, barn; CC8, conventional cage 8-tier; CC12, conventional cage 12-tier; EC8, enriched cage 8-tier; EC12, enriched cage 12-tier.

#### GP Center Gate Price and Retail Price

The GP center gate price was calculated by adding the GP center's expenses and profit to the farm gate price. The retail price or table egg price was calculated by adding the wholesaler or retailer profit margins (Table 8). According to the Food Price Trends Survey (MAFF, 2020a), the average table price of eggs was 207 yen per pack in 2020 (10 eggs of mixed sizes). In this study, the retail price of eggs was converted to a pack of 10 eggs, yielding values of CC8 = 247, CC12 = 247, EC8 = 281, EC12 = 281, AV = 372, and BR = 485 [yen/10 eggs]. The retail price of BR is approximately twice that of CC. This study calculated that the retail price of CC was higher than the statistics. In actual on-farm operations, the eggs produced in CCs become profitable by producing large quantities of eggs with a low-profit margin per egg. In this study, profit margins for farms, GP centers, wholesalers, and retailers were set at a uniform profit rate, without considering the differences in the production system. This is a possible reason for the discrepancy between the estimated and actual prices.

According to the Statistics Bureau of the Ministry of Internal Affairs and Communications (2022), the food cost for a household with 2.25 persons is estimated at 62,531 [yen/month]. Of that amount, eggs accounted for only 1.1% of food expenditure at 670 [yen/month]. When converted to the price of BR system eggs shown in this study, the price is 1,571 [yen/month], which is a higher 2.5% share of food expenditure. In addition, assuming that all of Japan's eggs were produced in the BR system, the soaring cost of eggs would likely affect the prices of factory eggs, such as liquid eggs and other products, thus affecting the prices of various food products.

On the other hand, the EC system has been evaluated as a system with the most economic benefits and removes many behavioral restrictions (Shimmura et al., 2007a, 2007b) Eggs are less expensive than beef, pork, and chicken, based on price-per-protein estimates (Tanabe, 1995), and from a nutritional perspective, eggs play a significant role. In this study, the table egg price of the EC system increased by less than that of the BR system and was only 1.4 times less than that of CC. In evaluating table egg prices, the EC is a realistic option for consumers. In addition, the level of hygiene required for egg production is very high in Japan, a country with a culture of raw food consumption. It has become clear that the condition of the hen house environment significantly affects the initial contamination of eggshells in terms of egg hygiene. It is important to control egg hygiene by minimizing dust in the hen house as much as possible, separating manure and eggs, and preventing them from engaging with each other (Kurihara et al., 1996); EC is considered superior in this respect. In addition, from the viewpoint of farm workers, EC systems can be operated with the same number of workers as CC. Consumers view the welfare of laying hens in a cage system as negative; the EC system has both improved animal welfare and economic advantages.

### Estimation of Table Egg Prices at Different Feed Grains

Table 10 shows the estimation of table egg prices for different feed grains. The retail price of eggs produced using domestic feed rice was the lowest. However, replacing corn with unhulled whole rice grain in diets for laying hens alters the sensory attributes of the eggs (Sasaki et al., 2019). Moreover, the yolk of eggs produced with feed rice turns white, and the albumen loses its elasticity (Tachikawa et al., 2009). It is generally recognized that the height of the albumen indicates the freshness of the eggs, and therefore, consumers must understand this food characteristic. The current high price of feed affects the use of domestic rice grain for livestock; however, producers and consumers need to understand the advantages and disadvantages associated with each feed. The retail price of imported non-GMO corn grain in a poultry system was calculated to be 52.6 yen per egg, the highest price estimated in this study.

**Table 10:** Estimation of table egg prices for different feed grains.

Category	Item	Unit	CC8	CC12	EC8	EC12	AV	BR
Retailer	Imported corn	yen/egg	24.7	24.7	28.1	28.1	37.3	48.5
	Domestic rice feed	yen/egg	24.4	24.4	27.8	27.8	37.0	48.2
	Domestic corn	yen/egg	25.5	25.5	29.0	29.0	38.3	49.7
	Imported non-GMO corn	yen/egg	27.6	27.6	31.1	31.1	40.7	52.6

Abbreviations: CC8, conventional cage 8-tier; CC12, conventional cage 12-tier; EC8, enriched cage 8-tier; EC12, enriched cage 12-tier; BR, barn.

## LIMITATIONS OF THE STUDY

To the best of our knowledge, only a dozen poultry farms have more than 100,000 birds per farm, and EC, AV, and BR facilities. Therefore, it was challenging to obtain average statistics from a small number of surveyed facilities because the number of target farms was small. Another problem faced was that, despite the anonymized publication of survey data, it was difficult to obtain production efficiency data, which is a trade secret. In the future, increasing the number of survey cases and measuring values from farm records to evaluate various management types may require further study. Despite these limitations, to our knowledge, this is the first study to focus on the differences in production costs and retail prices among farm animal welfare production systems in Japan.

## CONCLUSIONS

This study determined the production costs and retail prices of different poultry production systems. The results show that prices increase while implementing these systems. The study also revealed that the selection of the system among CC through BR significantly affects farmers, chicks brooding companies, and spent hen companies. For example, in terms of the cost of keeping chicks, it is difficult for breeders to invest in equipment corresponding to a non-cage system unless they can consistently sell the trained chicks to fly, and they have produced and maintained a high production rate. The spent hen company will need to revise their existing work procedures to capture the laying hen. Thus, changing production systems to AV and BR will have a significant impact. Farmers and other production stakeholders are unlikely to respond quickly to immediate changes. At the very least, it is essential to allow time for stakeholders to deal with disruption changes. While writing this paper, MAFF (2022b) started to collect for public comments about “Technical guidelines on feeding management for different livestock breeds (draft)” on the May 23, 2022. The demand for eggs from an improved animal welfare system will eventually increase to construct a sustainable production system. There are 2 ways to achieve this. One is a cage system with improved animal welfare and the other is a non-cage system. In any case, a farming system based on scientific evidence, such as animal behavior, economics, farmer welfare, food hygiene, food culture, and the climate of the production area must be constructed. It is necessary for the society as a whole to stimulate discussion on animal welfare in conjunction with the CC system through BR, deepen consumer understanding, and achieve a consensus on social policy decisions.

Although our data and study pertain specifically to Japan and the egg production situation there, rising costs of egg-laying production system considering animal welfare is a concern for many other countries as well. Our research may hold significance for decision-makers in countries where the shift toward animal welfare-based approach is taking place.
